# Cinefluoroscopy for assessment of mechanical heart valves with suspected dysfunction

**DOI:** 10.3389/fcvm.2022.952255

**Published:** 2022-09-06

**Authors:** Anselm A. Derda, Marvin M. Marquardt, Andreas Martens, Elion Mirena, Jens Vogel-Claussen, Tibor Kempf, Axel Haverich, Johann Bauersachs, L. Christian Napp

**Affiliations:** ^1^Department of Cardiology and Angiology, Hannover Medical School, Hannover, Germany; ^2^Department of Cardiothoracic, Transplant and Vascular Surgery, Hannover Medical School, Hannover, Germany; ^3^Institute for Diagnostic and Interventional Radiology, Hannover Medical School, Hannover, Germany

**Keywords:** mechanical heart valves, valvular heart disease, cinefluoroscopy, radiation, cath lab, cardiac surgery, heart failure

## Abstract

**Background:**

Mechanical heart valves (MHVs) are preferred prosthesis types in many, especially younger patients who need surgical valve replacement. Although echocardiography is most frequently performed for prosthesis assessment during follow-up, ultrasound artifacts usually preclude a precise investigation of prosthesis function. Cinefluoroscopy (CF) is a simple and effective method to analyze and quantify opening and closing of prosthesis leaflets but requires careful visualization of the valve using optimal viewing angles. Here, we investigated the quality of CF studies in clinical routine and their suitability for quantitative analysis of prosthesis function.

**Methods and results:**

We retrospectively identified 94 patients with 118 cinefluoroscopies performed by 31 different investigators in one tertiary center from 2012 to 2021. Of 150 MHVs (98% bi-leaflet prostheses), 87 (58%) were aortic, 53 (34%) mitral, 7 (5%) tricuspid, and 5 (3%) pulmonary valve prostheses, respectively. CF studies were categorized by their suitability to quantitatively assess opening and closing angles. Visualization of valve function was “sufficient” in 23%, “suboptimal” in 46%, and “unsuitable” in 31% of the cases.

**Conclusion:**

In clinical routine, only one-fourth of CF studies allow for a complete assessment of leaflet motion of MHVs. Although this may be in part due to the varying experience of operators, the high number of unsuitable studies suggests that optimal viewing angles may not be achievable in all patients. Further research is required to investigate standard viewing angles and anatomy after MHV implantation to improve the quality of CF studies and reduce radiation exposure of patients and operators.

## Introduction

The prevalence of valvular heart disease is steadily increasing, especially in industrialized countries (1). The leading cause is valvular degeneration, but endocarditis and congenital heart disease, such as bicuspid aortic valves, are also important (1). Although transcatheter valve therapy is rapidly evolving, surgical valve replacement is still required in many patients with severe stenosis or regurgitation (2). Especially in young and middle-aged patients undergoing surgery, mechanical heart valve (MHV) prostheses are preferred over biological prostheses because of their higher durability (3), and the standard prosthesis type is a bi-leaflet MHV (4). MHVs require lifelong oral anticoagulation (5) to prevent thrombus formation, which may result in embolism and life-threatening acute or subacute valve dysfunction (6). Although studies evaluating novel oral anticoagulants for MHVs are ongoing (7), vitamin K antagonists are the current standard for anticoagulation despite varying anticoagulation efficacy. In addition, there is a risk of pannus formation at various parts of the MHV, which may result in chronic dysfunction (8). Therefore, given the generally long durability of MHV prostheses, repetitive assessment of prosthesis function is necessary in many patients over time.

In cases of suspected MHV dysfunction, echocardiography allows for evaluation of valvular or paravalvular regurgitation, Doppler measurement of pressure gradients, and detection of larger thrombi, vegetations, or pannus (9). However, echocardiographic visualization of leaflets suffers from limited resolution and artifacts, and it is particularly unable to quantify leaflet motion of MHVs. In contrast, cinefluoroscopy (CF) is a non-invasive and quick method that allows for rather exact measurement of opening and closing angles of MHV leaflets (1). In this, CF is complementary to echocardiography and is considered a standard for assessing MHV function (10). Nevertheless, best practice recommendations for performing CF are not defined. CF critically depends on sufficient viewing angles to accurately acquire perpendicular cine views of the MHV with orthogonal visualization of both leaflets (10). Studies on the application of CF in clinical routine are lacking. Here, we questioned whether CF studies in daily clinical practice meet the procedural requirements for precise quantification of prosthesis function.

## Methods

The local patient data management system was retrospectively screened for CF studies on patients with MHVs. Cath lab records from 2012 to 2021 were systematically searched using the keywords [*klappenprothese] OR [mechanische] OR [mechan*] OR [mech*], which are German terms used to describe MHV. Only dedicated CF studies were included, and any studies performed for other reasons (such as angiographies) also showing MHV were excluded. In cases where the same patient had more than one CF study, only those performed by different investigators were considered. MHV types and manufacturers were identified from available data in the patient data management system. The study follows the principles outlined in the 1964 Declaration of Helsinki and later modifications, and was approved by the local ethics committee (10175_BO_K_2022).

CF scenes were categorized into three groups depending on visualization quality, independently by three investigators. A study was designated “sufficient” when the MHV ring was displayed at a perpendicular angle without tilting of the MHV ring or the leaflets. “Sufficient” therefore required sharp imaging of the leaflets to ensure an orthogonal view, which is necessary for reliable quantification of leaflet motion without parallax errors. CFs were “suboptimal” when there was either a non-perpendicular view of the ring or tilting of the leaflets. “Suboptimal” CFs were not sufficient for quantification of leaflet motion, since clinically approved solutions for quantifying leaflet motion from non-perpendicular views are not available. However, “suboptimal” CF studies still allowed for a meaningful qualitative assessment of leaflet opening and closing. The category “unsuitable” included all CFs in which MHV visualization precluded even qualitative judgment on the opening and closing of the leaflets. Mean gradients were taken from echocardiographic studies closely related to the time of CF. MHV function was reported as judged by the operator and confirmed by the study team. The number of acquired scenes and dose area products were compared using the Mann-Whitney test. A two-way ANOVA was conducted to compare the proportions of CF quality between valves. Calculations were performed using Prism 9 (GraphPad, United States) and STATA IC/16.1 (StataCorp LLC, United States).

CF studies were analyzed using IntelliSpace Cardiovascular V4.1 (Philips Medical Systems, Netherlands) or Centricity Enterprise Web V3.0 (GE Medical Systems, France).

## Results and discussion

From 2012 to 2021, 94 patients with 118 CF studies performed by 31 different investigators were identified (Table 1). The median number of acquired scenes was lower in patients with one MHV (4, range 1-11, *N* = 85) than in those with two or more MHVs (5, range 1-12, *N* = 33, *P* = 0.0009), which was associated with lower X-Ray dose area products (*P* < 0.0001). Of 150 different MHVs, 58% were in aortic, 34% in mitral, 5% in tricuspid, and 3% in pulmonary positions, respectively, and 98% of all MHVs were bi-leaflet prostheses (Table 1).

**TABLE 1:** Patient and cinefluoroscopy characteristics.

**Investigators - N**	31
**Patients - N**	94
Patients with one mechanical heart valve - N	72
Fluoroscopies - N	85
X-ray images, pts. with one valve - median (range)	4 (1-11)
Dose area product, one valve - mGy*cm^2^, median	3690 (96-38580)
(range) ^a^	
Patients with two or more mechanical heart valves - N	20
Fluoroscopies - N	33
X-ray images, pts. with two valves - median (range)	5 (1-12)
Dose area product, two valves - mGy*cm^2^, median	8361 (3,173-32,217)
(range) ^b^	
**Investigated valves total - N**	150
Bi-leaflet prosthesis - N (%)	147 (98%)
**Aortic - N (%)**	87 (58%)
Age - years, median (IQR)	54 (45-71)
Male sex - N (%)	46 (53%)
Date of surgery - year, median (range)	2005 (1971-2020)
Cine, years after surgery - median (range)	13 (0-50)
**Manufacturer - N (%)**	
St. Jude Medical	56 (64%)
Carbomedics	8 (9%)
On-X	3 (4%)
Medtronic	2 (2%)
ATS	1 (1%)
St. Jude Medical Conduit	1 (1%)
ATS Conduit	5 (6%)
Björk-Shiley prosthesis	1 (1%)
Unknown	10 (12%)
**Mitral - N (%)**	51 (34%)
Age - years, median (IQR)	53 (43-65)
Male sex - N (%)	22 (43%)
Date of surgery - year, median (range)	2007 (1988-2020)
Cine, years after surgery - median (range)	10 (0-30)
**Manufacturer - N (%)**	
St. Jude Medical	36 (71%)
Carbomedics	3 (6%)
On-X	2 (4%)
ATS	1 (2%)
Medtronic	1 (2%)
Björk-Shiley prosthesis	1 (2%)
Unknown	7 (13%)
**Tricuspid - N (%)**	7 (5%)
Age - years, median (IQR)	30 (29-39)
Male sex - N (%)	1 (14%)
Date of surgery - year, median (range)	2006 (2003-2013)
Cine, years after surgery - median (range)	11 (1-14)
**Manufacturer - N (%)**	
St. Jude Medical	7 (100%)
**Pulmonary - N (%)**	5 (3.2%)
Age - years, median (IQR)	41 (36-52)
Male sex - N (%)	2 (40%)
Date of surgery - year, median (range)	2007 (1985-2009)
Cine, years after surgery - median (range)	10 (9-33)
**Manufacturer - N (%)**	
St. Jude Medical	4 (80%)
Björk-Shiley prosthesis	1 (10%)

^a^Data available for 68 fluoroscopies, ^b^data available for 24 fluoroscopies, and pts., patients.

**FIGURE 1:**
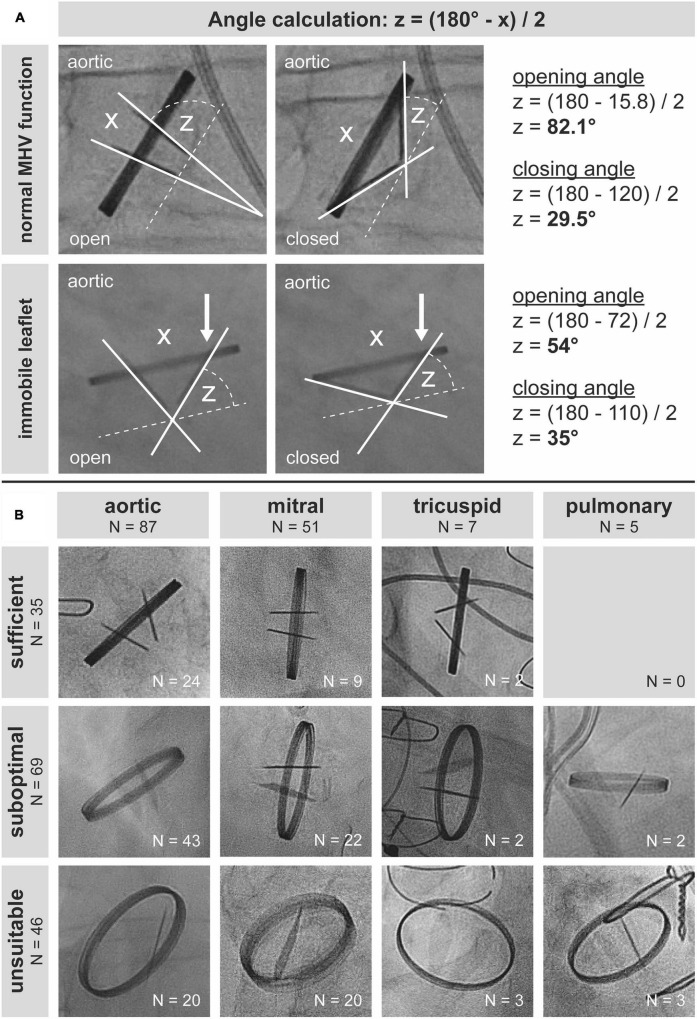
Best practice and real-world practice of cinefluoroscopy of mechanical heart valves (MHVs). (A) Evaluation of leaflet function in two patients with a mechanical aortic valve prosthesis. Orthogonal visualization of leaflets with a perpendicular view of the ring without any parallax or oblique viewing is a prerequisite for accurate quantification of opening and closing angles. (B) Of 150 cinefluoroscopic valve investigations, only 35 allowed for quantification of opening and closing angles.

For sufficient assessment of MHV function, the ring should be visualized in a perpendicular projection without parallax, and the entire range of leaflet motion should be assessable with sharply imaged leaflets to avoid erroneous measurement of angles (Figure 1A). This allows for calculating opening and closing angles (two examples in Figure 1A), in order to estimate potential dysfunction. Of note, the calculated opening angle of a valve in a CF study is equal to the sum of the opening angles of both leaflets: Symmetric impairment of opening of both leaflets by 10° and reduction of opening of one leaflet by 20° finally results in the same opening angle. This underlines the clinical importance of quantitative and not only qualitative assessment of leaflet motion during CF.

Of 150 MHV studies, 35 (23.3%) were technically sufficient to quantify leaflet motion. Sixty-nine (46%) studies were suboptimal, though allowing for qualitative assessment of leaflet motion at least to some extent. Forty-six (30.7%) studies were unsuitable for assessing MHV function (Figure 1B). Proportions of CF quality did not differ (*P* = 0.249) between MHVs in aortic (sufficient 27.6%, suboptimal 49.4%, unsuitable 23%), mitral (17.7, 43.1, 39.2%), tricuspid (28.6, 28.6, 42.9%), and pulmonary (0, 40, 60%) position, respectively. In our cohort, there was no pulmonary MHV with sufficient visualization, which may be due to the generally low number of pulmonary MHVs.

**FIGURE 2:**
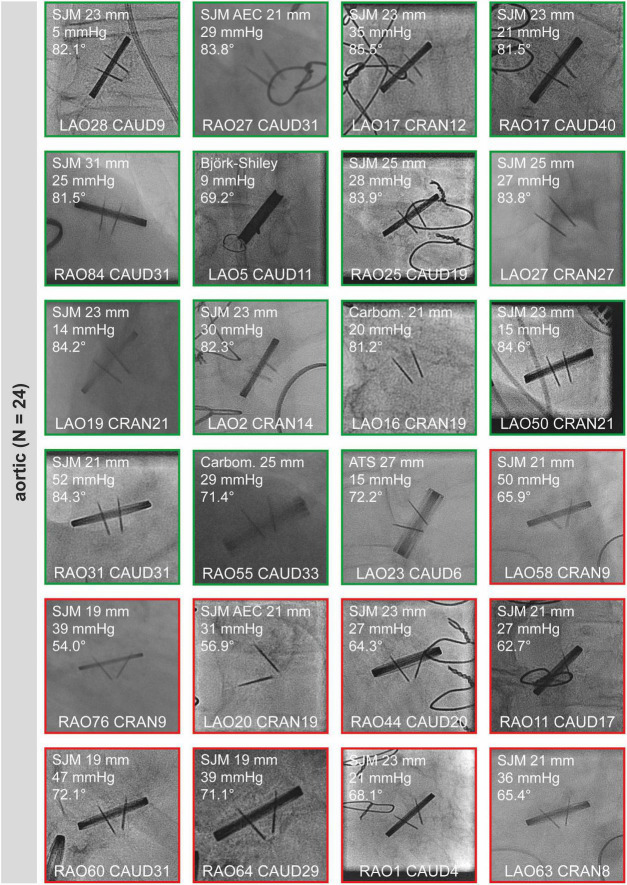
Sufficient cinefluoroscopy studies of MHVs in aortic position. MHVs had either normal leaflet opening and closing (green) or leaflet dysfunction (red). MHVs are shown during maximum opening. Individual images show the opening angle (for details refer to Figure 1), mean gradient from transthoracic echocardiography, and projection angles in CF. For comparison, the mean opening angles of SJM, ATS, and Carbomedics MHVs are 83.4, 69.5, and 77.1°, respectively (12). Some images are already shown in Figure 1. AEC, aortic valve extended cuff; ATS, ATS Medical; Carbom., Carbomedics; CAUD, caudal; CRAN, cranial; LAO, left anterior oblique; MHV, mechanical heart valve; RAO, right anterior oblique; SJM, St. Jude Medical.

**FIGURE 3:**
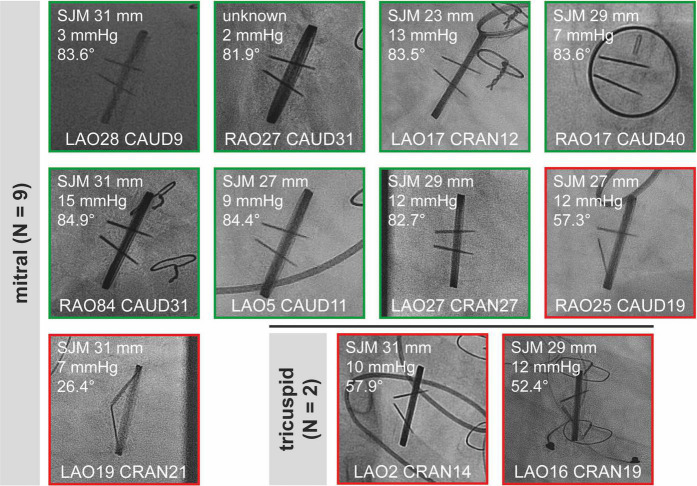
Sufficient cinefluoroscopy studies of MHVs in mitral and tricuspid position. MHVs had either normal leaflet opening and closing (green) or leaflet dysfunction (red). MHVs are shown during maximum opening. Individual images show the opening angle (for details refer to Figure 1), mean gradient from transthoracic echocardiography, and projection angles in CF. For comparison, the mean opening angles of SJM, ATS, and Carbomedics MHVs are 83.4, 69.5, and 77.1°, respectively (12). Some images are already shown in Figure 1. CAUD, caudal; CRAN, cranial; LAO, left anterior oblique; MHV, mechanical heart valve; RAO, right anterior oblique; SJM, St. Jude Medical.

Snapshots of all “sufficient” CF studies are shown in Figure 2 (aortic valves) and Figure 3 (mitral and tricuspid valves). Median opening angles were much lower in patients with MHV dysfunction (median, 62.7°; range, 26.4°-72.1°) than in those with normally functioning valves (median, 83.6°; range, 71.4°−85.5°, *p* < 0.0001) (Figures 2, 3 and Supplementary Figure 1). The spectrum of angulations required to visualize a given MHV was very wide, ranging from far right anterior oblique caudal to far left anterior oblique cranial (Figures 2, 3). There was no correlation between mean gradients in transthoracic echocardiography and the presence of MHV dysfunction (Supplementary Figure 1), likely because gradients are influenced by more factors than leaflet function such as patient-prosthesis-mismatch, hypertrophy, and others.

This study is the first to investigate the imaging quality of MHVs by CF in clinical practice. Over a period of 10 years, CF studies allowed for quantitative assessment of only 23.3% and qualitative evaluation of an additional 46% of 150 investigated MHVs, respectively. The range of acquired scenes was 1 to 12 per procedure, and CF was performed ranging from 0 to 50 years after surgery.

Previous studies have demonstrated that CF is superior and complementary to echocardiography for assessing the full range of leaflet motion (10, 11). Accordingly, CF is recommended by current guidelines as a standard tool for diagnosing prosthesis dysfunction (1). In our cohort, only one-fourth of the CF studies was sufficient for quantitative evaluation of MHV function. Furthermore, given that in theory only one scene per MHV is necessary for quantitative and qualitative assessment of prosthesis function, most CF procedures contained too many acquisitions.

One potential reason for the results of this study is a lack of knowledge and education. Operators with limited experience might tolerate suboptimal results when leaflet motion is visible in an insufficient projection. Importantly, manufacturers’ values for leaflet opening and closing angles differ between prosthesis types, and real-world opening angles of normally functioning MHVs are in part different from angles provided by the manufacturers (12). Nevertheless, the calculation of angles is an important tool for longitudinal assessment of MHV function, such as during treatment of thrombosis or for evaluation of chronic pannus formation. Therefore, quantitative evaluation of MHV function is essential during CF. Although there is consensus that a perpendicular view of the ring with tilt-free visualization of leaflet motion is required, there is no further best practice recommendation for performing CF. Moreover, it is unclear whether there is an optimal starting angle when selecting the viewing angle during CF. The examination mainly depends on the experience and self-set standards of the investigators. This likely results in increased radiation exposure of the patient and the operator (13).

Second, we assume that CF is not able to visualize all MHVs in a perpendicular tilt-free projection, as suggested by the wide spectrum of angulations needed for sufficient CF in our study cohort. Contemporary cath labs still have limited viewing angles, and in some patients, the anatomy of the valvular annulus plane may preclude optimal CF of the MHV (14). Consistently, it has been demonstrated that not all parts of the coronary arterial tree can be visualized by cardiac catheterization in a perpendicular orthogonal view in all patients (15). Moreover, positioning of the prosthesis during surgery or altered geometry by the procedure may play a role in visualization during CF (16). Therefore, research on the anatomy after valve replacement surgery is needed to better understand the optimal viewing angles of MHVs.

## Conclusion

In clinical practice, only a minor proportion of CF studies meets the requirements for both quantitative and qualitative assessments of MHV function. This may be in part due to limited operator experience, but the high number of unsuitable studies suggests that sufficient viewing angles may not be achievable in all patients. Further research is required to test whether standard viewing angles can be defined and generate best practice recommendations for CF, reduce radiation exposure of patients and operators, and improve the quality of CF studies.

## Data availability statement

The datasets presented in this article are not readily available because local regulations preclude data transfer. Requests to access the datasets should be directed to LCN, napp.christian@mh-hannover.de.

## Ethics statement

The studies involving human participants were reviewed and approved by Ethikkommission der Medizinischen Hochschule Hannover. Written informed consent for participation was not required for this study in accordance with national legislation and institutional requirements.

## Author contributions

AAD, MMM, EM, and LCN analyzed the images. AAD, MMM, and LCN analyzed the data. AAD, MMM, EM, AM, and LCN interpreted the data for the study. MMM and LCN prepared the figures. AAD, MMM, and LCN drafted the manuscript. AAD, MMM, AM, JV-C, JB, and LCN revised the manuscript. LCN designed and supervised the research. All authors contributed to the article and approved the final version of the manuscript.

## Abbreviations

CF: = cinefluoroscopy
IQR: = interquartile range
MHV: = mechanical heart valve
N: = number.

## References

[1] Vahanian A Beyersdorf F Praz F Milojevic M Baldus S Bauersachs J 2021 ESC/EACTS guidelines for the management of valvular heart disease. *Eur Heart J.* (2022) 43:561–632. 10.1093/eurheartj/ehab395 34453165

[2] Beckmann A Meyer R Lewandowski J Markewitz A Gummert J. German heart surgery report 2020: The annual updated registry of the German society for thoracic and cardiovascular surgery. *Thorac Cardiovasc Surg.* (2021) 69:294–307. 10.1055/s-0041-1730374 34176107

[3] Dunning J Gao H Chambers J Moat N Murphy G Pagano D Aortic valve surgery: Marked increases in volume and significant decreases in mechanical valve use–an analysis of 41,227 patients over 5 years from the society for cardiothoracic surgery in great Britain and Ireland national database. *J Thorac Cardiovasc Surg.* (2011) 142:776–782.e3. 10.1016/j.jtcvs.2011.04.048 21924147

[4] Gopal S Hauser JM Mahboobi SK. *Mechanical aortic valve replacement.* Tampa, FL: StatPearls (2022). 33232009

[5] Goldstone AB Chiu P Baiocchi M Lingala B Patrick WL Fischbein MP Mechanical or biologic prostheses for aortic-valve and mitral-valve replacement. *N Engl J Med.* (2017) 377:1847–57. 10.1056/NEJMoa1613792 29117490 PMC9856242

[6] Lancellotti P Pibarot P Chambers J Edvardsen T Delgado V Dulgheru R Recommendations for the imaging assessment of prosthetic heart valves: A report from the European association of cardiovascular imaging endorsed by the Chinese society of echocardiography, the inter-American society of echocardiography, and the Brazilian department of cardiovascular imaging. *Eur Heart J Cardiovasc Imaging.* (2016) 17:589–90. 10.1093/ehjci/jew025 27143783

[7] Jawitz OK Wang TY Lopes RD Chavez A Boyer B Kim H Rationale and design of PROACT Xa: A randomized, multicenter, open-label, clinical trial to evaluate the efficacy and safety of apixaban versus warfarin in patients with a mechanical On-X aortic heart valve. *Am Heart J.* (2020) 227:91–9. 10.1016/j.ahj.2020.06.014 32693197 PMC7484170

[8] Ma WG Hou B Abdurusul A Gong DX Tang Y Chang Q Dysfunction of mechanical heart valve prosthesis: Experience with surgical management in 48 patients. *J Thorac Dis.* (2015) 7:2321–9. 10.3978/j.issn.2072-1439.2015.12.25 26793354 PMC4703666

[9] Zoghbi WA Chambers JB Dumesnil JG Foster E Gottdiener JS Grayburn PA Recommendations for evaluation of prosthetic valves with echocardiography and doppler ultrasound: A report from the American society of echocardiography’s guidelines and standards committee and the task force on prosthetic valves, developed in conjunction with the American college of cardiology cardiovascular imaging committee, cardiac imaging committee of the American heart association, the European association of echocardiography, a registered branch of the European society of cardiology, the Japanese society of echocardiography and the Canadian society of echocardiography, endorsed by the American college of cardiology foundation, American heart association, European association of echocardiography, a registered branch of the European society of Cardiology, the Japanese society of echocardiography, and Canadian society of echocardiography. *J Am Soc Echocardiogr.* (2009) 22:975–1014; quiz82–4. 10.1016/j.echo.2009.07.013 19733789

[10] Cianciulli TE Lax JA Beck MA Cerruti FE Gigena GE Saccheri MC Cinefluoroscopic assessment of mechanical disc prostheses: Its value as a complementary method to echocardiography. *J Heart Valve Dis.* (2005) 14:664–73. 16245506

[11] Khouzam RN. Cinefluoroscopy as the gold standard for mechanical valve mobility. *Can J Cardiol.* (2007) 23:998. 10.1016/s0828-282x(07)70865-6 PMC2651426 17932579

[12] Suh YJ Kim YJ Hong YJ Lee HJ Hur J Im DJ Measurement of opening and closing angles of aortic valve prostheses in vivo using dual-source computed tomography: Comparison with those of manufacturers’ in 10 different types. *Korean J Radiol.* (2015) 16:1012–23. 10.3348/kjr.2015.16.5.1012 26356549 PMC4559772

[13] Picano E Vano E. The radiation issue in cardiology: The time for action is now. *Cardiovasc Ultrasound.* (2011) 9:35. 10.1186/1476-7120-9-35 22104562 PMC3256101

[14] De Paulis R Salica A. Surgical anatomy of the aortic valve and root-implications for valve repair. *Ann Cardiothorac Surg.* (2019) 8:313–21. 10.21037/acs.2019.04.16 31240175 PMC6562095

[15] Kocka V Theriault-Lauzier P Xiong TY Ben-Shoshan J Petr R Labos M Optimal fluoroscopic projections of coronary Ostia and bifurcations defined by computed tomographic coronary angiography. *JACC Cardiovasc Interv.* (2020) 13:2560–70. 10.1016/j.jcin.2020.06.042 33153569

[16] Rosner A Avenarius D Malm S Iqbal A Schirmer H Bijnens B Changes in right ventricular shape and deformation following coronary artery bypass surgery-insights from echocardiography with strain rate and magnetic resonance imaging. *Echocardiography.* (2015) 32:1809–20. 10.1111/echo.12973 26010320

