# Supplementary material for: Cinefluoroscopy for assessment of mechanical heart valves with suspected dysfunction
Source: Front Cardiovasc Med. 2022 Sep 6;9:952255. doi: 10.3389/fcvm.2022.952255 (PMC9486207; doi:10.3389/fcvm.2022.952255)
Supplement: Supplementary file 1 [file Data_Sheet_1.docx]

**Supplemental Figure 1. Opening angles and mean pressure gradients of mechanical heart valves (MHVs) with sufficient cinefluoroscopic visualization.** MHVs without dysfunction are shown in green, and those with dysfunction are shown in red. Left: MHVs in aortic position, right: MHVs in mitral and tricuspid position. There is no statistically significant difference between mean pressure gradients in dysfunctional MHVs as compared to those without dysfunction (aortic: *p*=0.1904, mitral/tricuspid: *p*=0.8152). Mean pressure gradients were taken from echocardiographic studies closely related to the time of cinefluoroscopy. MHV function was reported as judged by the operator and confirmed by the study team.
