# Supplementary figures and images for: Cinefluoroscopy for assessment of mechanical heart valves with suspected dysfunction
Source: Front Cardiovasc Med. 2022 Sep 6;9:952255. doi: 10.3389/fcvm.2022.952255 (PMC9486207; doi:10.3389/fcvm.2022.952255)

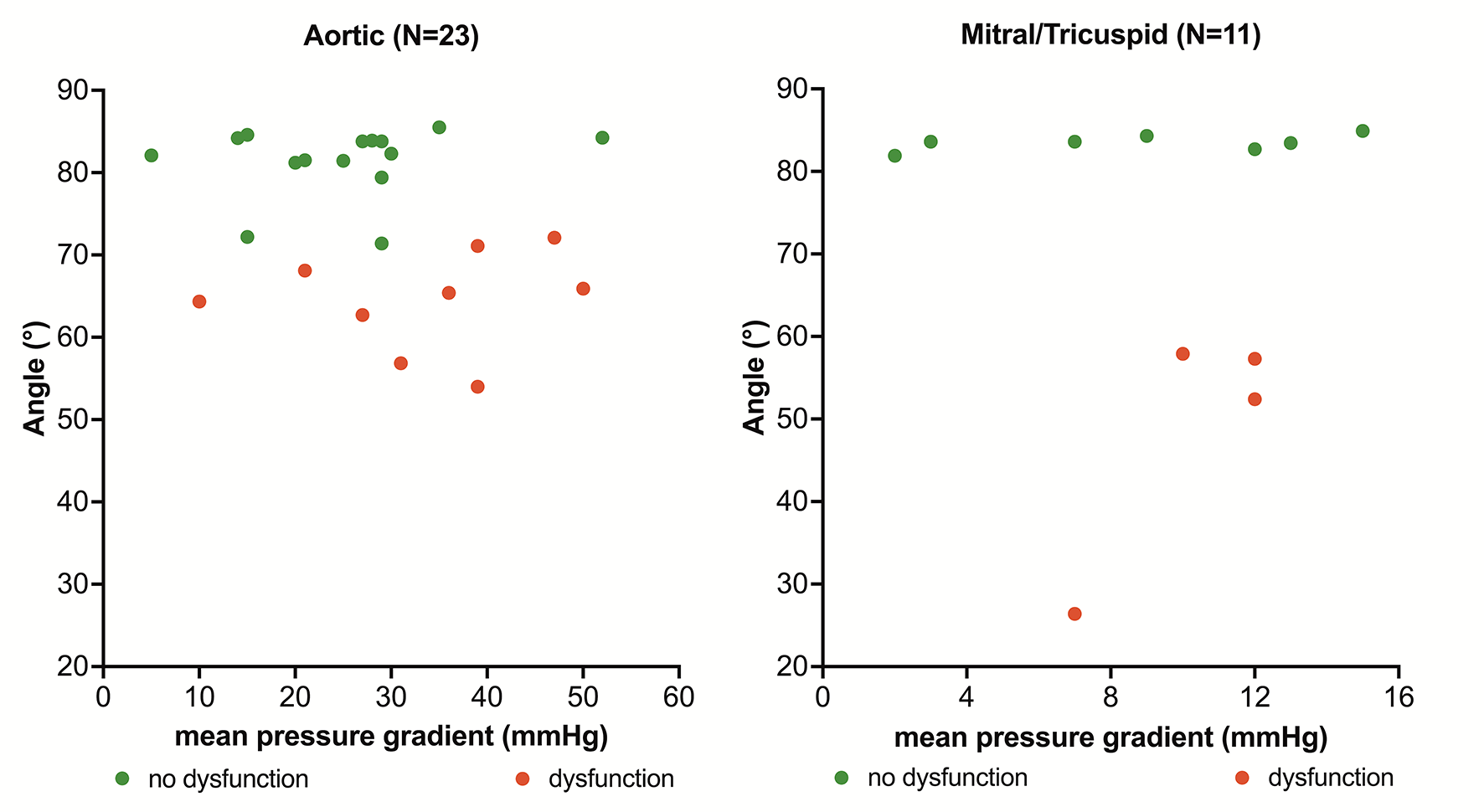

Supplement: Supplementary file 2 [file Image_1.tiff]
